# Periosteal pressure sensitivity of the chest bone as a measure for autonomic function in ischemic heart disease

**DOI:** 10.3389/fnins.2025.1574942

**Published:** 2025-05-21

**Authors:** Nanna Ørsted, Søren Ballegaard, Jesper Kristiansen, Finn Gyntelberg, Ake Hjalmarson, Jens Faber

**Affiliations:** ^1^Department of Chemistry, Technical University of Denmark, Kongens Lyngby, Denmark; ^2^Ballegaard Ltd. Copenhagen, Skodsborg, Denmark; ^3^The National Research Centre for the Working Environment, Copenhagen, Denmark; ^4^Department of Cardiology, Sahlgrenska University Hospital, Gothenburg, Sweden; ^5^Department of Endocrinology, Herlev University Hospital, Herlev, Denmark; ^6^Faculty of Health and Medical Sciences, Copenhagen University, Copenhagen, Denmark

**Keywords:** autonomic function, Baroreflex-mediated cardiovascular response, resilience, Betablockade medication, heart rate variability, periosteal pressure sensitivity, tilt table Test1

## Abstract

**Purpose:**

In 177 patients with ischemic heart disease and elevated periosteal pain sensitivity of the chest bone indicative of autonomic nervous system dysfunction, we test the hypotheses, (i) there is an association between the tilt table responses for the baroreflex-mediated cardiovascular response heart rate variability and periosteal pain sensitivity of the chest bone, (ii) these responses are affected differently by use of beta blockade medication, and (iii) reduction of an elevated periosteal pain sensitivity of the chest bone, during three months of non-pharmacological intervention, improves these responses to tilt table testing.

**Results:**

Baroreflex-mediated cardiovascular response, heart rate variability and periosteal pain sensitivity measures all changed significantly in response to tilt table test but only periosteal pain sensitivity and baroreflex-mediated cardiovascular responses were internally associated. Use of beta blockade medication inhibited the baroreflex-mediated cardiovascular response and heart rate variability responses but did not of periosteal pain sensitivity. In response to three months intervention with the aim to reduce the elevated periosteal pressure pain, all responses to tilt table test improved, but for the baroreflex-mediated cardiovascular response and heart variability in non-users of beta blockade, only. Participants who achieved a predefined minimum reduction of 15 units in periosteal pain sensitivity demonstrated significant improvement when compared to participants did not obtain this reduction.

**Conclusion:**

Periosteal pressure sensitivity of the chest bone at rest as well as the response to tilt table test seem new and promising measures of autonomic nervous system dysfunction, which remains unaffected by BB medication.

## Introduction

The autonomic nervous system (ANS) enables many bodily functions to maintain homeostasis (Goldstein, 2019). When a pathophysiological challenge becomes too great, a vicious circle develops in the shape of dysfunctional ANS (ANSD) that itself is disease promoting (Goldstein, 2019). This is a potential risk of worsening for a wide range of diseases in which ANSD is prevalent including diabetes, heart disease, and cancer (Bergmann et al., 2014; Faber et al., 2021; Khandelwal et al., 2023). Despite this, no evidence-based treatment of ANSD has been established, partly due to lack of consensus regarding measurement of ANSD. At present, measures of ANS function and ANSD are sensitive to a combination of autonomic tests that monitor multiple peripheral autonomic functions (Novak, 2011). Among these tests, the Tilt Table Test (TTT) that records baroreflex-mediated cardiovascular response (Jaradeh and Prieto, 2003; Freeman and Chapleau, 2013), and the Heart Rate Variability (HRV) measure that reflects cardiovascular autonomic function, have become widely used as ANS function tests (Brinza et al., 2021). HRV is typically measured by either beat-to-beat variation or by power spectral analysis of the electrocardiogram (Carstensen et al., 2011; Fleischer, 2012; Karemaker, 2017).

ANSD may involve either sympathetic dominance/hyperactivity or parasympathetic hypoactivity (Cygankiewicz and Zareba, 2013). The ANS system is physiologically a hierarchical system, anatomically consisting of: (i) brain regulating centers including insular cortex and hypothalamus, (ii) brainstem, and (iii) the peripheral sympathetic and vagal nerves (Benarroch, 2020). The baroreflex-mediated cardiovascular response and HRV tests reflect ANS function and are under the influence of beta-adrenergic receptor antagonists (beta-blockers, BB) inhibiting the efferent sympathetic autonomic activity (Feldman et al., 2010; Billman, 2013; Gordan et al., 2015). The fact that many patients with ischemic heart disease (IHD) receive treatment with BB warrants the use of a measure of ANS function that is not affected by BB.

Measures of the periosteal pressure sensitivity (PPS) at the sternum repeatedly demonstrated an association with levels of ANS function and ANSD in healthy control subjects (Ballegaard et al., 2009) as well as in patients with ischemic heart disease (IHD) (Ballegaard et al., 2015; Ballegaard et al., 2016), or type 2 diabetes (T2D) (Faber et al., 2021). The association is evident from comparisons of PPS values to tests of autonomic reflexes such as the withdrawal reflex eyeblink (Ballegaard et al., 2009), resting heart rate (HR) (Faber et al., 2021; Ballegaard et al., 2009), responses of systolic blood pressure (SBP), HR and Pressure-Rate Product (PRP) to dynamic testing by TTT (Ballegaard et al., 2015), HRV as measured by beat-to-beat variation during or Stand-Up Test in T2D (Faber et al., 2021), and to autonomic homeostatic regulation of glucose metabolism (Goldstein, 2019).

The PPS values appear to remain unaffected by BB medication in contrast to the baroreflex-mediated cardiovascular response and HRV (Faber et al., 2021; Ballegaard et al., 2016). This observation gave rise to the hypothesis that the PPS may be regulated in centers of the brain, that are insensitive to the activity of beta-adrenergic neurotransmission. As such, the orexin cell system in the lateral part of the hypothalamus might be the central PPS regulating center (Faber et al., 2021).

In three consecutive RCT’s in healthy people (Ballegaard et al., 2014), in people with ischemic heart disease (Bergmann et al., 2014), and in people with Type 2 diabetes (Faber et al., 2021), it has been found that reduction of an elevated PPS is associated with concomitant reductions in elevated health risk factors reflecting ANSD, as these factors are controlled by the ANS. These factors include blood pressure, heart rate and work of the heart expressed as the Pressure-rate Product, total cholesterol and LDL cholesterol in healthy persons (Ballegaard et al., 2014), response to tilt table test and overall survival in persons with ischemic heart disease (Ballegaard et al., 2015; Ballegaard et al., 2016; Ballegaard et al., 2023), and in people with the type 2 diabetes blood levels of glycated hemoglobin and as well as homeostatic regulation of glycated hemoglobin (Faber et al., 2021; Mistry et al., 2022; Thayer et al., 2010). In line with this notion, a recent editorial suggests PPS as a target measure for ANSD (Dekker et al., 2000).

Furthermore, in people with IHD, the sensory nerve stimulation was found to be able to alleviate an anginal attack in association with an acute (1-min) reduction of an elevated PPS (Ballegaard et al., 2023). This observation is addressed as a research question by observing the one-minute changes of PPS and the baroreflex-mediated cardiovascular response to TTT.

Hypothesizing PPS as an indicator of ANSD, we tested the following hypotheses, and studied individuals with stable IHD undergoing a TTT as the stimulus of the ANS before and after 3 months intervention, aiming at reducing resting PPS:

1. The responses to TTT are internally associated for the three measures (i.e., PPS; HRV and Baroreflex-mediated cardiovascular response) of ANS function; however, differently affected by beta blockade medication.
2. Reversal of ANSD, measured as reduction of an elevated resting PPS during three months of intervention, is associated with an improvement in all three measures for ANS function.

## Methods

### Design and participants

The present study evaluated the dynamic ANS response as measured in people with stable IHD and measured one and eight minutes after TTT on three different responses, the HRV, the baroreflex-mediated cardiovascular response, and PPS. These measured were recorded before and after three months of follow-up. The study group comprised all participants in a randomized controlled trial (RCT) originally performed to evaluate the effect on depression score, degree of persistent stress, and quality of life induced by a non-pharmacological intervention aiming at reducing ANSD, measured as resting PPS (Bergmann et al., 2014). Thus, due to the nature of the non-pharmacological intervention, active and passive intervention groups could be pooled, when evaluating the response to a TTT. In line with previous RCT’s we used a pre-study definition of a minimum relevant reduction of an elevated PPS to be 15 PPS units, reflecting a 50% increase in pressure threshold (Faber et al., 2021; Ballegaard et al., 2015; Thayer et al., 2010). This made it possible to distinguish between participants who at 3 months evaluation demonstrated a clinically relevant reduction in PPS (i.e., ≥ 15 PPS unit reduction), designated reverters, and those who did not obtain such reduction, designated non-reverters. Finally, the effect of non-use versus use of BB was evaluated.

**Figure 1:**
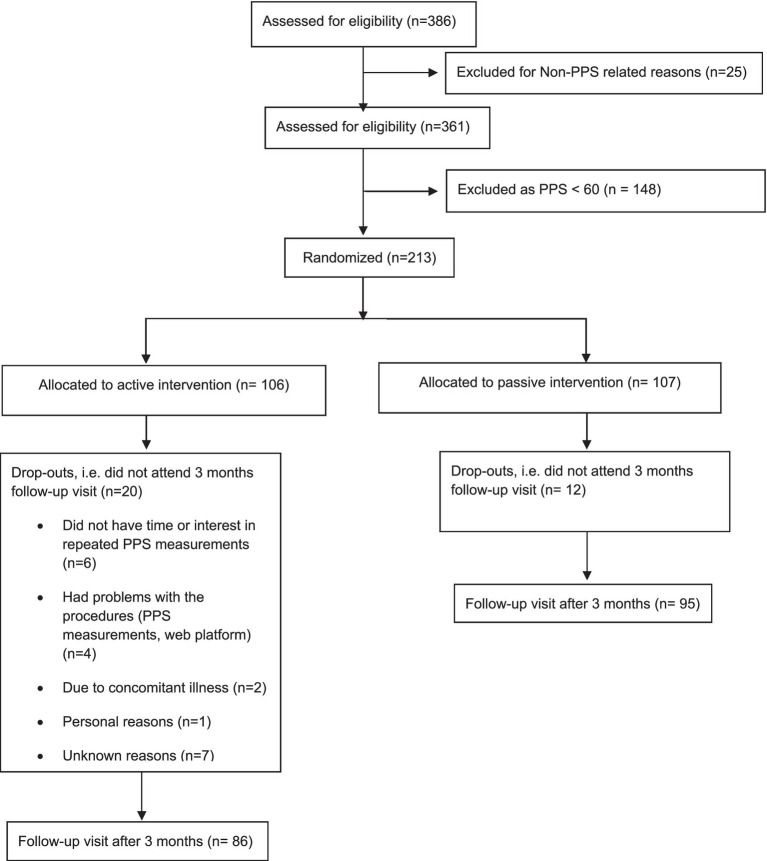
CONSORT diagram (Bergmann et al., 2014).

As depicted from Figure 1 (CONSORT diagram), 361 patients with stable IHD participated in a cross-sectional study evaluating the possible association between PPS and questionnaires covering depression, persistent stress and quality of life (Bergmann et al., 2013). Those people (*N* = 213) having elevated resting PPS (≥ 60 units), indicative of persistent stress and ANSD, were then randomized 1:1 to either active intervention for 3 months or passive control (Bergmann et al., 2014). In 181 of the 213 participants of the RCT a TTT evaluating dynamic changes in the baroreflex-mediated cardiovascular response, HRV and PPS were obtained both before and after the 3 months follow-up (86 participants in the active group, and 95 in the control group) (Ballegaard et al., 2015). Some of the results, 8–minute recordings on PPS and baroreflex-mediated cardiovascular response, have been published previously (Ballegaard et al., 2016). The present sub-study expands this experimental study by comparing the results from pre-defined analyses of HRV, the baroreflex-mediated cardiovascular response and the PPS response to a TTT.

Regarding the influence of BB medication, the participants were divided into groups of non-users and users. All beta-adrenergic medications used among the participants in this study were beta^1^ –adrenergic blockade medications. Among the users, four individuals used hydrophilic beta-blockers (e.g., Atenolol), which fails to pass the blood–brain barrier, and the remaining 102 individuals used lipophilic beta blockers that possess such penetrative property. To exclude the potential source of bias, the four individuals using Atenolol were excluded. This leaves two groups of participants; non-users (*N* = 75) and users (*N* = 102) (Ballegaard et al., 2016).

Informed consent was obtained from all the participants after providing oral and written information about the study. The study was approved by the local ethics committee (ID H–4 –2010–135) and was registered on www.clinicaltrials.gov (NCT01513824).

### Interventions

Due to the complex nature of the non-pharmacological treatment, we have chosen to present the full version of the program as previously published (Ballegaard et al., 2023):

All participants completed cardiac rehabilitation more than six months prior to inclusion. Upon inclusion into the RCT, all participants, active as well as passive intervention group members received the information that the level of persistent stress was elevated, as a sign of poor cardiovascular health. Both groups received an 80-page manual of general stress management from the perspective that persistent stress negatively affects IHD. All medication of active and passive group members remained unchanged during the last month prior to the baseline examination, and all participants received instructions not to change medication during the initial 3-month period of participation. Thereafter, medication was administered by the general practitioner. The interventions included no new medication (Table 1).

**Table 1:** Baseline demographics according to the study groups in the intervention RCT: non-users or users of beta-blockade medication (−/+ Beta-blockage).

General information	– Beta-blockade	+ Beta-blockade
*N*	75	102
Male, %	66	78*
Age in years, mean (SD)	62 (9)	63 (7)
MDI, (arbitrary units), mean (SD)	9.4 (6.8)	8.6 (7.5)
Cardiac variables		
Previous myocardial infarction (%)	63	68
Treated with PCI (%)	64	73
Treated with CABG (%)	23	31
Cardiac risk factors		
Body Mass Index, (kg/m^2^), mean (SD)	27.4 (4.8)	27.6 (4.6)
Triglyceride, (mmol/l), mean (SD)	1.3 (0.8)	1.5 (0.9)
Total Cholesterol, (mmol/l), mean (SD)	4.4 (1.0)	4.3 (1.0)
HDL Cholesterol, (mmol/l), mean (SD)	1.3 (0.4)	1.2 (0.4)
LDL Cholesterol, (mmol/l), mean (SD)	2.5 (0.8)	2.3 (0.8)
Current smoker (%)	2	2
Self-reported co-morbidity		
Heart failure (%)	19	44***
Chronic obstructive lung disease (%)	5	9
Diabetes (%)	12	15
Previous stroke (%)	6	8
Previous treatment for depression (%)	16	14
Medication		
Cholesterol-lowering medication (%)	85	96
Calcium antagonists (%)	19	25
Angiotensin-II antagonist and/or ACE inhibitors (%)	51	59
Diuretics (thiazide or furosemide) (%)	33	35

For between-group significance: * *p* < 0.05; ** *p* < 0.001; *** *p* < 0.0001.

Active intervention group members underwent a specific 3-month educational program of non-pharmacological self-care with assignments from a personal instructor. The education had two elements, a preventive part aimed at the reduction of elevated sympathetic activity (ANSD), believed to be measurable as elevated resting PPS, and an active intervention part aimed at an *ad hoc* reduction of acutely elevated PPS, intended to alleviate attacks of angina pectoris. The preventive part included the following:

1. Mandatory daily PPS measurements at home with instruction of how to perform PPS measurements, including a guideline for interpretation of the PPS measure, how to reflect on the measure, and a guide to clinical signs of alarm that require immediate attention.
2. Mandatory daily cutaneous sensory nerve stimulation at specific sites on the body surface aimed at a reduction in elevated baseline PPS values and subsequent maintenance of low resting PPS.
3. Daily recording of PPS measures in a web journal as a personal guide to the effect of the intervention, with *ad hoc* cognitive reflection in cases of sudden elevations of the PPS measure.
4. Ongoing professional surveillance based on a personal web journal allowing pro-active professional intervention in cases of missing or deviating PPS measurements.
5. A range of free-choice mental and physical exercises presented in the book of general stress management aimed at reducing stress in support of persistent lowering of resting PPS.

At the onset of active intervention, active group subjects learned by personal one-to-one instruction to identify tender spots on the chest bone (intended as a sign of an acutely elevated sympathetic activity); to apply moderate pressure with a finger at one of these locations, preferably the most tender one, without causing pain; and to maintain the pressure for 30–60 s until a reduction of the tenderness at the cutaneous pressure point. In participants with cases of angina pectoris, we expected to observe a concomitant subsidence of the angina pectoris attack. If not, we instructed the patient to take nitroglycerin. We interpret a marked reduction of tenderness at the cutaneous pressure point within the first minute of stimulation as evidence of correctly applied pressure, predicted to reduce elevated sympathetic activity. Without a reduction, the subject repeats the procedure at another tender skin surface point in the proximity. We instructed a spouse in daily cutaneous nerve stimulation at the back of the chest of the subject as a preventive measure, including ad hoc measures in cases of present angina. All participants received information on how to conduct nerve stimulation on the back by themselves (e.g., using a small firm ball in a long stocking, with a knot on each side of the ball, for applying pressure against a wall) as an alternative or supplement to the nerve stimulation conducted by a spouse. Active intervention group members received a 40-page booklet with instructions into the program, as well as a quick guide card meant to always be available with general instructions on how to alleviate an attack of angina pectoris.

Passive intervention group members continued the cardiac rehabilitation program initiated at least six months prior to the inclusion in the RCT. As the active group members, at the baseline examination, they received the information that their level of persistent stress was elevated as a sign of poor cardiovascular health, and they received the same 80-page manual of general stress with management suggested from the perspective that persistent stress negatively affects IHD. Thereafter, the passive group members received no further intervention-related instructions or intervention-related contact.

### Measures of TTT response

Baseline values as well as response to TTT were recorded in (i) HRV, using the four variables (HRV-Total Power (TP), HRV-High Frequency band (HF) and HRV-Low Frequency band (LF), and HRV-LF/HF band ratio); (ii) baroreflex-mediated cardiovascular response, using heart rate (HR), systolic blood pressure (SBP), work of the heart calculated as SBP x HR (PRP); and (iii) PPS. Both TTT responses after one and 8 min were measured. However, since the calculation of spectral components of HRV requires that the ECG recording be at least 5 min long HRV responses to TTT could not be calculated after one minute of TTT; but only after 8-min TTT. The variables were recorded before and after 3 months. Changes over time were calculated as post-intervention values (i.e., after 3 months) minus before values, thus resulting in negative values if a variable was reduced during the 3 months, and positive values if the variable increased.

For the matter of clarity before (baseline) responses to TTT are named “delta,” whereas changes in this response to the TTT over the 3 months of study period are named “delta–delta.”

#### Pressure pain sensitivity (PPS measurement)

An algometric instrument (StressMeter: Ballegaard (Carstensen et al., 2011) Stresscare, Skodsborg Strandvej 198, 2,942 Skodsborg Denmark) (Patent No EP 1750772 B1) was used for the measurement of the Periosteal Pressure Sensitivity of the sternum (PPS). The instrument measures the pressure sensitivity or threshold, which is transformed into a logarithmic scale and inverted into a sensitivity scale from 30 to 100 arbitrary units; that is: A high PPS value indicates high sensitivity and thus a low pressure threshold. For analysis, the mean of two consecutive recordings was used. If the difference then was more than 10 units, a third measurement was performed, and the mean of all three recordings was used.

#### Tilt table test (TTT)

This test induces a transient decrease in para-sympathetic tone followed by an increase in sympathetic tone and is usually conducted for the diagnosis with respect to sympathetic dominance, i.e., ANSD (Cheshire and Goldstein, 2019; Aponte-Becerra and Novak, 2021).

We used the technique as suggested by Novak (2011), with punctual measurement of BP, HR and PPS. Due to the nature of the PPS measurement, this measurement can only be conducted a few times within a short observation period to ensure that the repeated pressure does not affect the measure significantly. From clinical experience, the maximum number of PPS measurements were sat to four; two measurements at the end of the 10 min resting period, and third and fourth measurement after 1 and 8 min of tilting, respectively. Blood pressure and heart rate were measured at the same time intervals and right before the PPS measurement. As such, Heart-rate variability parameters (HRV), baroreflex-mediated cardiovascular response (HR, SBP and PRP), and PPS were measured 4 times: After a 10-min rest in the supine position, two measurements were conducted (the mean representing resting values). Then the participant was passively tilted to an angle of 70 degrees. A third set of measurements was conducted, approximately one minute after the initiation of the tilt, which takes less than 20 s. In this position the participant was left for 7 min, after which a fourth set of measurements was performed (i.e., the 8–minute response). The difference between the third measurement and resting value represents the 1-min response and the difference between the fourth measurement and resting values the 8–minute response.

#### Heart rate variability (HRV)

Four HRV variables were included, for which the consensus in terms of interpretation was strongest: The HRV-Total Power and HRV-HF are considered mainly to reflect the parasympathetic tone (Electrophysiology, Task Force of the European Society of Cardiology the North American Society of Pacing, 1996; Berntson et al., 1997), and the HRV-LF reflecting both the cardiac sympathetic and parasympathetic tone. The calculated HRV-LF/HRV-HF Band ratio increases with increasing sympathetic activity/dominance and/or reduced parasympathetic activity (Cygankiewicz and Zareba, 2013).

An electrocardiogram (ECG) was recorded using a 3-lead Lifecard CF Holter Monitors (Del Mar Reynolds Medical, Inc., Irvine, CA, USA). ECG recording segments were sampled during two standardized conditions with the patient on the tilt table: During the last 5 min of the 10 min rest in supine position and during the last 5 min of the 7 min with the patient in 70 degrees tilt. Artefacts and non-normal beats in the ECG segments were autodetected by a commercial software (Impresario version 2.8, Del Mar Reynolds Medical Inc., Hertford, UK). Each 5 min ECG segment was inspected visually, and undetected artefacts were marked and removed before the HRV analysis. The electrocardiograms (ECGs) were sampled with a sampling frequency of 128 Hz. To calculate the heart inter-beat interval series, the ECGs were processed as described previously (Kristiansen et al., 2011). First, the fiducial point of each R-peak was determined after cubic-spline interpolation to 512 Hz. Next, the RR-intervals were filtered for possible outliers (ectopic beats, falsely detected beats, missed beats, etc.). Finally, the RR-intervals were resampled with a frequency of 4 Hz and linearly detrended. The spectral components of the HRV for 5-min segments of the RR-interval series were estimated by Welch’s averaged, modified periodogram method (Hamming window size 256 points, 50% overlap). The low-frequency power (HRV-LF) was calculated for the frequency range 0.04–0.15 Hz and high-frequency power (HRV-HF) in the range 0.15–0.4 Hz (Electrophysiology, Task Force of the European Society of Cardiology the North American Society of Pacing, 1996).

#### Systolic blood pressure (SBP), heart rate (HR) and pressure-rate-product (PRP)

Blood pressure and heart rate were recorded using a Thuasne automatic blood pressure monitor (W0840 002001, Microlife ref. BP-#AA1-2, BP 243–92,307), Levaillois-Perret Cedex, France. For the analysis the mean of two measurements was used. If the between-measurement difference was more than 10%, a third measurement was carried out and the result was calculated as the mean of the three recordings.

### Statistics

Non-parametric statistics were used for group comparison due to a non-normal data distribution, Wilcoxon two-sample test for between-group analysis, Mann–Whitney one-sample test for with-in group analysis. For correlation analysis, the Pearson test for linear parametric correlation analysis was used assuming normality in continuous variables. For testing statistical significance, all randomized participants who concluded the second set of measurements were pooled (*n* = 177). Statistical testing for group differences in response to TTT (Tables 2, 3, 4): mixed model regression with group (beta-blocker usage), gender, age, and baseline HRV level as independent variables.

**Table 2:** Baseline characteristics for the effect values with respect to resting values, and responses to 8-min tilt table testing according to the study groups in the intervention RCT: Non-users or users of beta blockade medication (−/+ Beta blockage).

Resting values	– Beta-blockade	+ Beta-blockade
Resting PPS, mean (SD)	76.6 (13.0)	78.3 (13.0)
Resting pulse, mean (SD)	64 (11)	59 (9) c
SBP, mean (SD)	134 (16)	133 (17)
Diastolic blood pressure, mean (SD)	78 (9)	80 (9)
Pressure-rate Product	8,690 (1,981)	7,938 (1,728) c
HRV-TP band	7.05 (0.95)	6.70 (1.05) c
HRV-LF band	5.72 (1.12)	5.32 (1.24) c
HRV-HF band	5.27 (1.37)	4.78 (1.34) c
HRV-LF/HF ratio	0.46 (0.94)	0.53 (0.97)
Responses to 8-min tilt table testing (TTT)		
PPS response to TTT, mean (SD)	−5.4 (11.6) ***	−4.7 (14.2) **
Systolic blood pressure (SBP) response to TTT, mean (SD)	- 3.3 (13.1)	−2.3 (12.3)
Heart rate (HR) response to TTT, mean (SD)	8.4 (7.1) ***	6.1 (5.8) *** c
Pressure-rate Product (PRP) response to TTT, mean (SD)	−42 (1011)	- 279 (1236) *
HRV-TP band response to TTT, mean (SD)	−0.20 (1.2)	−0.19 (0.9)
HRV-LF band response to TTT, mean (SD)	−0.23 (1.2)	−0.20 (1.0)
HRV-HF band response to TTT, mean (SD)	−0.82 (1.4) ***	−0.85 (1.0) ***
HRV-LF/HF ratio response to TTT, mean (SD)	0.59 (1.0) ***	0.66 (1.1) ***

For response to TTT: * *P* < 0.05; ** *P* < 0.01; ***P < 0.001 ^c^ Significant difference between user and non-user of beta-blocker, *p* ≤ 0.05.

Units: PPS: arbitrary units (au); Systolic blood pressure (SBP): mmHg; Heart rate (HR): beats/min; Pressure rate product (PRP): mmHg x beats/min; HRV-Total power (HRV-TP): ln (ms-2) HRV-low frequency power (HRV-LF): ln (ms-2); HRV-high frequency power (HRV-HF): ln (ms-2); HRV-LF/HF-band ratio: no unit. HRV variables were obtained from the spectral components for 5-min segments of the RR-interval series.

**Table 3:** Changes in physiological responses to passive tilt (after 1 and 8 min at 70°) in ischemic heart disease patients during 3 months of follow-up for PPS reverters (i.e., resting PPS reduction during intervention period ≥ 15 au) comparing BB non-users (*N* = 30) with BB users (*N* = 46), For the overview, significant between-group differences are marked with green color.

TTT response after:	Physiological response	Changes in TTT response during follow-up period for PPS reverters (follow-up TTT response—baseline TTT response). Delta-delta values Grouped as BB non-user and BB users	
		Non-user (*N* = 30)	User (*N* = 46)
	PPS (SD)	6.7 (14.9) *	14.2 (14.2) ***
	SBP (SD)	3.8 (10.6) ^c^	−3.4 (11.8) ^c^
1 min	HR (SD)	−0.4 (4.9)	1.0 (5.5)
	PRP (SD)	303.3 (1038.1)	−92.2 (839.3)
	PPS (SD)	8.2 (14.6) * ^c^	14.5 (14.6) *^c^
	SBP (SD)	7.9 (14.6) * ^c^	0.2 (15.2) ^c^
	HR (SD)	−0.4 (5.9)	0.8 (6.1)
	PRP (SD)	188.3 (1323.5)	235.2 (1474.3)
8 min	HRV-TP (SD)	−0.8 (1.0) ** ^c^	−0.01 (1.0) ^c^
	HRV-LF (SD)	−1.01 (1.5) * ^c^	−0.06 (1.1) ^c^
	HRV-HF (SD)	−0.59 (1.1) ^c^	0.03 (1.0) ^c^
	HRV-LF/HF (SD)	−0.42 (1.1)	−0.09 (0.9)

*** Significant different from zero, *P* < 0.001; * *p* < 0.05. ^c^ Significant difference between user and non-user of beta-blocker, *P* ≤ 0.05.

PPS: arbitrary units (au); Systolic blood pressure (SBP): mmHg; Heart rate (HR): beats/min; Pressure rate product (PRP): mmHg x beats/min; HRV-Total power (HRV-TP): ln (ms-2) HRV-low frequency power (HRV-LF): ln (ms-2); HRV-high frequency power (HRV-HF): ln (ms-2); HRV-LF/HF-band ratio: no unit. HRV variables were obtained from the spectral components for 5-min segments of the RR-interval series.

**Table 4:** Changes for TTT responses for 3 months comparing PPS reverters and PPS non-verters.

Physiological variables at tilt table testing	Changes in tilt table response for PPS reverters versus PPS non-reverters		Cohen’s effect size
	PPS reverters	PPS non-reverters	
			
PPS (mean, SD) (All subjects: *N* = 70/96)	11.8 (16.8)^***,a^	−3.6 (16.2) ^*,a^	0.84
SBP (mean, SD) (BB non-users, only: *N* = 30/41)	7.9 (14.6) ^**,b^	−3.3 (13.7) ^b^	0.54
HRV-LF Band (mean, SD) (BB non-users, only: *N* = 13/26)	−1.01 (1.47) ^*,b^	−0.17 (0.69) ^b^	−0.89

Regarding HRV and Baroreflex responses, only BB non-users were included. Regarding PPS: both BB users and BB non-users are included.

Significantly different from zero, ***p < 0.001. ** *p* < 0.01. * *p* < 0.05. Significant difference between PPS reverters and PPS non-reverters, ^a^ *P* < 0.001; ^b^ *P* < 0.01.

Units: PPS: arbitrary units (au); Systolic blood pressure (SBP): mmHg; Heart rate (HR): beats/min; Pressure rate product (PRP): mmHg x beats/min; HRV-Total power (HRV-TP): ln (ms^−2^) HRV-low frequency power (HRV-LF): ln (ms^−2^); HRV-high frequency power (HRV-HF): ln (ms^−2^); HRV-LF/HF-band ratio: no unit. HRV variables were obtained from the spectral components for 5-min segments of the RR-interval series.

Brown and Prescott (2014). Change in response to TTT during the follow-up period (delta–delta) is presented in absolute values and as percentage change calculated as change during the 3 months follow-up period (i.e., 3 months values minus before (baseline) values; that is a negative result means a reduction of the outcome measure) divided by change during before (baseline) TTT multiplied by 100 (%).

The statistical program, SPSS, version 18 (SPSS Inc., Chicago, Illinois, USA) was used for all analyses.

We used Cohen’s effect size as a supplementary assessment of the clinical effect. We calculated the Cohen effect sizes by analysis of covariance (ANCOVA), which uses the post-test as the outcome and adjusts for baseline (pre-test) scores. We did so to minimizes bias from regression toward the mean and to account for baseline differences (Mistry et al., 2022). In relation to clinical significance, a Cohen effect size of less than 0.2 represents a minor clinical effect, 0.2–0.4 a small effect, 0.4–0.7 a moderate effect, and ≥ 0.7 a large effect (Baer, 2010).

#### Subgroup analysis

“The analysis of hypothesis 2 focused on PPS reverters (participants achieving ≥15 PPS unit reduction) to evaluate whether ANSD reversal, as defined by PPS reduction, translates to improvements in baroreflex-mediated cardiovascular response and HRV dynamics. This subgroup analysis aligns with the study’s mechanistic aim to test the physiological link between PPS reduction and ANS function.”

The reverter/non-reverter classification was pre-defined based on prior RCTs (Faber et al., 2021; Ballegaard et al., 2015; Thayer et al., 2010) where a ≥15 PPS unit reduction was clinically meaningful and correlated with improved cardiovascular outcomes. This threshold ensures a homogeneous population for evaluating ANSD reversal."

## Results

### Demographic data

Table 1 shows the demographic data for the 177 participants of the RCT, divided in the group of BB non-users (*N* = 75) and users (*N* = 102). Only number of participants with heart failure was different, being highest in BB users.

### Baseline measurement (before follow-up period)

**Figure 2:**
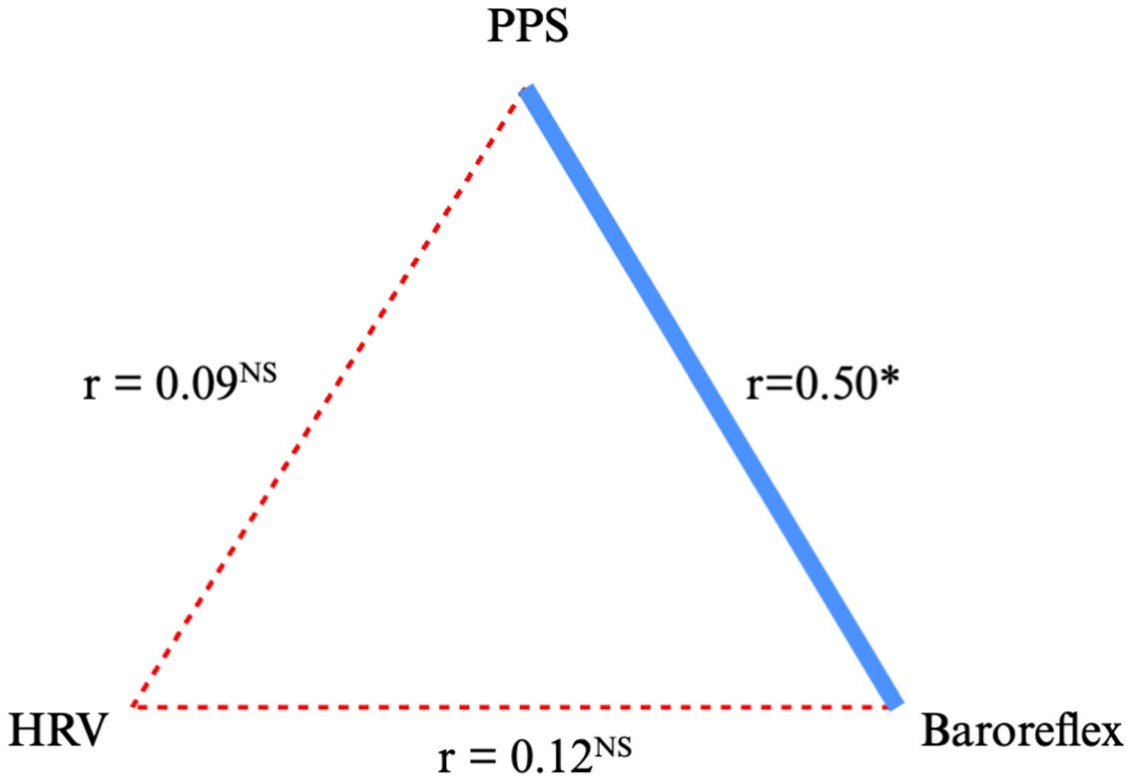
The correlations between HRV, baroreflex-mediated cardiovascular response and PPS response to TTT at baseline. Data from Table 2 are used and only for the group of non-users of BB. The correlation coefficients shown (*r*-values) are the mean numerically *r*-value for the four HRV parameters: TP, LF, HF and LF/HF ratio, for the three Baroreflex-mediated cardiovascular measures: SBP, HR, and PRP, and for PPS *r*-value. * All *p* < 0.01; NS, not significant.

*A:* The baseline measurements (Tables 2, 5 and Figure 2).

**Table 5:** Baseline correlations for non-users/users (*N* = 75/102) of Beta blockade medication.

BB non-users/ users	HRV-TP	HRV-LF	HRV-HF	HRV-LF/HF	PPS
SBP	0.16/ 0.05	0.18/ 0.13	0.20*/ 0.03	−0.06/ 0.07	0.52**/ 0.39***
HR	−0.20*/ 0.06	−0.21*/ 0.04	0.28**/ 0.16	0.11/ 0.19*	0.53**/ 0.46***
PPR	0.00/ −0.01	−0.03/ 0.08	0.02/ −0.05	−0.06/ 0.11	0.44**/ 0.29***
HRV-TP					0.10/ 0.00
HRV-LF					0.12/ 0.06
HRV-HF					0.05/ 0.03
HRV-LF/HF					0.08/ 0.02

Physiological responses to passive tilt at baseline (after 8 min at 70°) in ischemic heart disease patients. For a graphic presentation among the group of non-BB users (*N* = 75), see Figure 2. ^*^ *P* = < 0.05; ** *P* < 0.01, ^***^ *p* < 0.0001.

Units: PPS: arbitrary units (au); Systolic blood pressure (SBP): mmHg; Heart rate (HR): beats/min.

Pressure rate product (PRP): mmHg x beats/min; HRV-total Power: (HRV-TP): LnTP (ms^−2^); HRV-low frequency power (HRV-LF): ln (ms^−2^); HRV-high frequency power (HRV-HF): ln (ms^−2^): HRV-LF band HRV-HF ratio (HRV-LF/HF): no unit HRV variables were obtained from the spectral components for 5-min segments of the RR-interval series.

Resting values at baseline and before tilting, and when comparing BB non-users with BB users, the group of BB non-users had higher HR, PRP, HRV- TP band, HRV-LF band and HRV-HF band. All *p* < 0.05 (Table 2).

Regarding the baseline TTT responses (8-min value minus resting value before tilting) of Baroreflex-mediated cardiovascular response, HRV and PPS, the responses were significant and alike for BB non-users and BB users for PPS, heart rate, HRV-LF/HF ratio and HRV-HF band (all *p* < 0.05) (Table 2), and only significantly different between BB non-user and users for HR. Combining the two groups (*N* = 177), all tested variables reacted significantly to TTT (data not shown).

There was a close and positive correlation between PPS response to TTT on the one side, and TTT responses of SBP, HR and PRP on the other side (all *p* < 0.0001). In contrast, PPS did not correlate to any of the four HRV variables. The correlations between HRV and baroreflex-mediated cardiovascular responses were present, however inconsistent, and weak, and consistently significantly weaker than the corresponding ones between PPS and baroreflex-mediated cardiovascular responses (all between-group *p* < 0.001) (Table 5 and Figure 2).

### B: changes during follow up period

**Figure 3:**
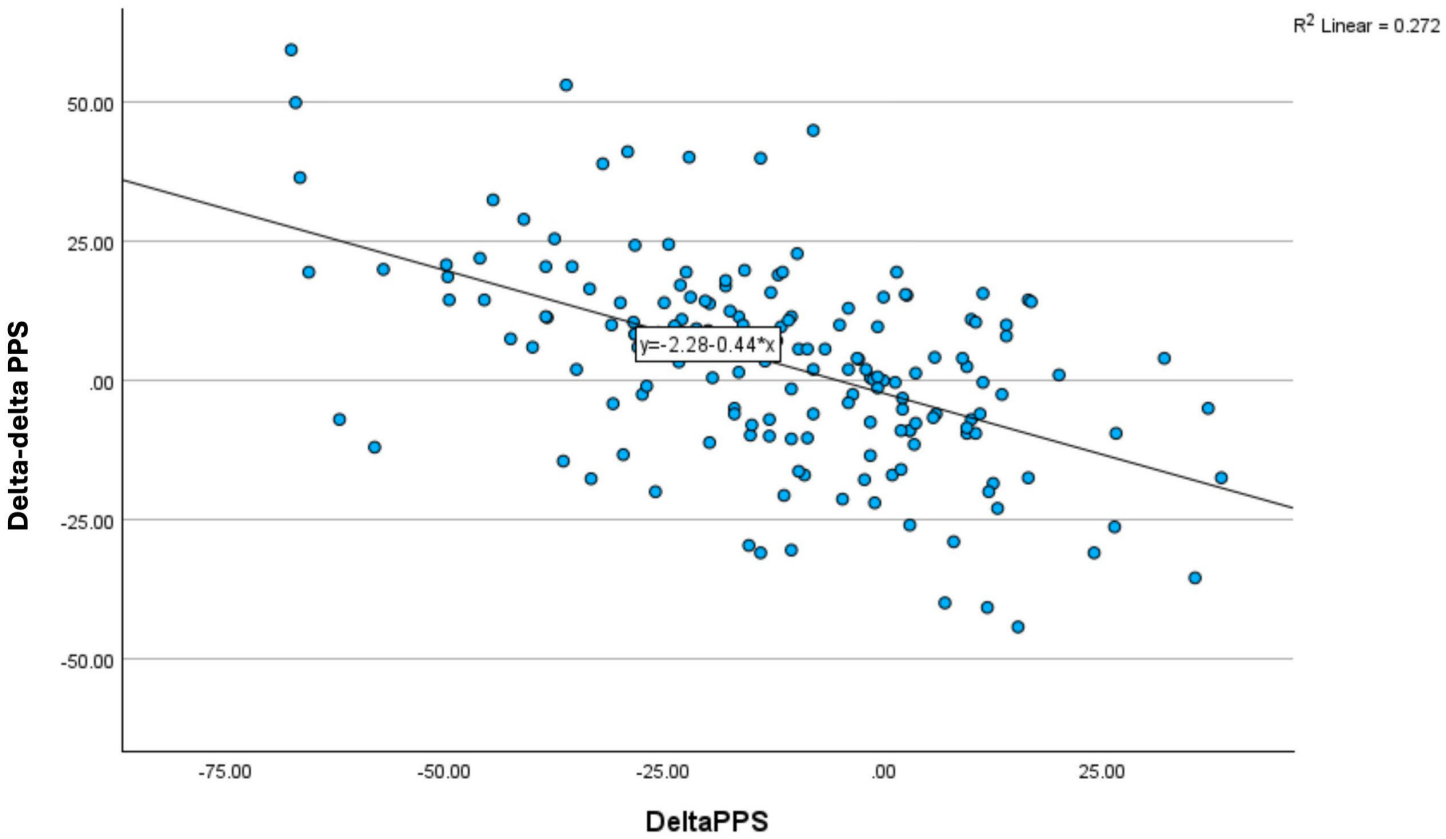
Changes in 8-min response TTT for PPS (Delta–deltaPPS, y-axis) during 3 months of non-pharmacological intervention showing the association to change in resting PPS (DeltaPPS, x-axis) for non-users of Beta blockade medication (*N* = 170, *r* = − 0.52, *p* < 0.001).

During the intervention period, resting PPS decreased significantly in the active intervention group from mean 81 to 58 (*p* < 0.0001), and significantly more that the passive intervention group (mean changes from 81 to 72) (Bergmann et al., 2014). According to the hypothesis of the study, this reduction was the premise for evaluating the changes in TTT responses. For PPS reverters and PPS non-reverters, the PPS decreased significantly from mean 81 to 50 in the PPS reverter group, while in contrast PPS increased significantly in the PPS non-reverter group, from mean 76 to 78 (*p* > 0.01) (Figure 3).

*B1:* Associations between changes in resting PPS, and changes in TTT responses during follow up period.

**Figure 4:**
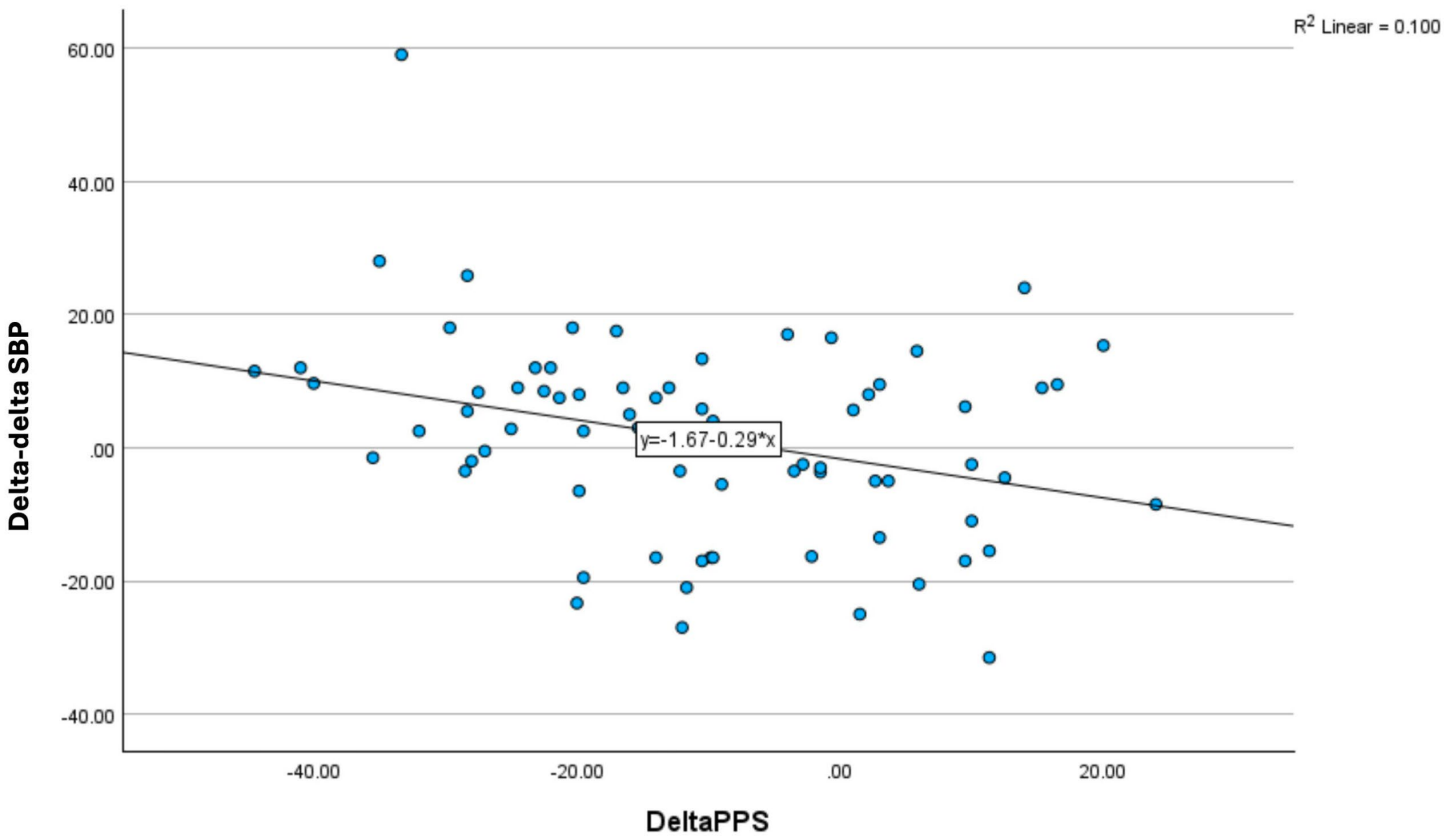
Changes in 8-min response TTT for systolic blood pressure (Delta–deltaSBP, y-axis) during 3 months of non-pharmacological intervention showing the association to change in resting PPS (DeltaPPS; x-axis) for non-users of Beta blockade medication (*N* = 71, *r* = − 0.32, *p* = 0.007).

The reduction in resting PPS was significantly correlated to an increase in PPS response to TTT during intervention period (delta–delta) (*r* = 0.52, *p* < 0.0001) (*N* = 177) as previously published (Ballegaard et al., 2015) (Figure 4).

When taking the use of BB into account, changes in resting PPS during 3 months of intervention period was significantly correlated to change in SBP response to TTT (delta–delta) (*r* = − 0.32; *p* = 0.007) (*N* = 75) for the group of BB non-users (Figure 3), however absent for the group of BB user (*r* = 0.13; *p* > 0.1) (*N* = 102) (between group *p* = 0.04). This means that the greater the decrease in resting PPS the greater the SBP response to tilting as observed over 3 months, but only in BB non-users. In contrast, the association between reduction in resting PPS during the intervention period and the change in PPS response to TTT was significant for BB users (*r* = −0.58) and BB non-users (*r* = −0.37) alike (both *p* < 0.01).

There were no significant correlations between changes in resting PPS during the intervention and changes in the four HRV responses to TTT (all *p* > 0.1).

*B2:* Changes in 8-minute responses to TTT after three months compared to before (baseline) responses to TTT.

The PPS and PRP responses to TTT both increased significantly, while none of the remaining variables changed significantly. When taken the use of BB medication into consideration (non-users versus users), this did not affect the PPS and PRP changes, but for the HRV responses, the changes were significant for 2 out of 4 variables in the BB non-user group only, and with a significant between-group difference, when non-users and users were compared. The changes were of substantial size; the PPS response increased + 3 PPS units (60%) (*N* = 177) (*p* < 0.05) and the PRP response increased 240 mmHg x beats per minute (122%) (*N* = 177) (*p* < 0.05) and among the BB non-users %, the HRV-TP band decreased 0.4 (200%) (*N* = 75) (*p* > 0.05) and the HRV-LF band decreased by 0.45 (196%) (*N* = 75) (*p* < 0.05), and for both HRV variables with a significant between-group difference, when BB non-users were compared to BB non-users (both *p* < 0.05).

*B3:* Changes in 8-minute responses to TTT after 3 months of follow up compared to before (baseline) responses to TTT for PPS reverters versus PPS non-reverters (Table 3 and Figure 4).

Similarly to the findings with respect to resting PPS, there was a significantly different pattern of PPS response to TTT during the 3 months of follow-up between PPS reverters (*N* = 76) and PPS non-reverters (*N* = 101); There was a significant increase among reverters (mean increase 11.8 units; 236%) (*p* < 0.001) compared to a significant decrease among non-reverters (mean decrease: 3.4 units; 68%) (*p* < 0.05), and with a between-group significance (*p* < 0.001). For the HRV-LF band, there was also a significant between-group difference comparing reverters (190% decrease) and non-reverters (30% decrease) (*p* < 0.05). None of the other variables showed a significant between-group difference.

Among PPS reverters, and when taken the use of BB into account, the PPS responses increased significantly in BB non-users (*N* = 30) as well as in BB users (*N* = 46) and with no between-group difference. In contrast, the SBP response to TTT increased significantly by 7.9 mmHg (240%) (*N* = 30) at the follow-up TTT among BB non-users, compared to 0.2 mmHg (*N* = 46) (between-group *p* < 0.05) For the HRV variables, the mean HRV-LF band responses to TTT decreased significantly during the follow-up period for BB non-users (505% decrease), compared to BB users (32% decrease) (between-group *p* > 0.05). A similar pattern was observed for HRV-TP band; 421% decrease in BB non-users and 5% decrease in BB users (*p* < 0.05) A non-significant 72% decrease in the TTT response was seen for the HRV-HF band among BB non-users, which however was significantly different from BB users (4% increase) (between-group *p* < 0.05).

When comparing PPS reverters and PPS non-reverters, and taking usage of BB into account for the SBP and HRV-LF band responses to TTT, concomitant and substantial changes were observed for the PPS reverter group in the TTT responses during the intervention period for three measures alike (i.e., PPS, SBP and HRV-LF) (Table 2 and Figure 4). However, there were no significant internal correlations between the three set of changes (all *p* > 0.1) (Figure 4). Figure 3 shows the TTT responses for the three measures in percentage comparing PPS reverters and PPS non-reverters, including the lack of effect for the latter group.

*B4:* Changes in 1-minute responses to TTT after 3 months of follow up compared to before (baseline) responses to TTT for PPS reverters versus PPS-non-reverters, including sub-group analysis on Beta-blockade medication, non-users versus users (Table 3).

At baseline, the TTT response for HR, PRP and PPS changes significantly and coherently.

There was a significantly different pattern of PPS response to TTT during the 3 months of observation between PPS reverters (increase: 267%) and PPS non-reverters (decrease: 52%), both being within-group significant as well as being between-group significant.

When taken BB usage into account among PPS reverters, the PPS responses increased for both groups, and with no between-group difference. In contrast, the SBP response to TTT increased by 3.8 mm Hg (115% increase) in BB non-users, while it decreased by 3.4 mm Hg (148% decrease) in BB users (between-group *p* < 0.05).

*C:* Active versus passive intervention.

Regarding the effect of the intervention program, which was tested in the RCT, the probability of being a PPS reverter was 4.1 times higher in the active versus the passive intervention group, when using per protocol analysis (*p* < 0.0001) and 3.1 (*p* = 0.0001) when using intention-to-treat analysis.

Changes during the 3 months intervention period, and when active and passive intervention were compared and using per protocol analyses, showed that mean (SD) resting PPS changed by −20.5 (22.2) (*N* = 84) in active group compared to – 4.3 (16.9) (*N* = 93) in the passive intervention group (*p* < 0.0001). For changes in PPS response to tilting, the corresponding figures were + 5.0 (18.9) and + 1.1 (17.1) for active and passive group, respectively (NS). For changes in Systolic Blood pressure and HRV-LF band responses, and among non-users of BB, the corresponding changes were: + 4.2 mmHg (15.7) (*N* = 32) versus −0.9 mmHg (14.3) (*N* = 39) and – 0.51 (1.4) versus – 0.4 (0.8), respectively (both NS)

*D:* Clinical relevance.

Table 4 shows the changes in TTT during the three months of intervention comparing PPS reverters versus PPS non-reverters for the PPS, SBP and HRV-LF band. For all three the between-group differences were significant and regarding the potential clinical relevance, Cohen’s effect sizes were 0.8 for PPS, 0.5 for SBP and 0.9 for the HRV-LF band.

## Discussion

Main findings, according to the two hypotheses tested, were: In a population of people with stable IHD and elevated resting PPS, indicating ANSD, we found that:

Regarding hypothesis no. 1: At baseline, using the TTT experimental set-up for dynamics of ANS function, the three measures HRV, Baroreflex-mediated cardiovascular response and PPS, all showed significant changes after 8-min of TTT. However, the only consistent association between the measured variables was between the baroreflex-mediated cardiovascular response and the PPS response (Table 5 and Figure 2).

The effect of beta blockage medication on the response to baseline TTT was pronounced on the HRV and baroreflex-mediated cardiovascular responses to TTT, however absent with respect to the PPS response to TTT. This absence confirms other findings (Faber et al., 2021; Ballegaard et al., 2016) and may suggest that PPS is controlled by control centers of ANS which are located higher in the ANS hierarchy than the brainstem, and which are unaffected by beta blockade medication. The most likely candidate may be the orexin system of the lateral hypothalamus (Faber et al., 2021).

Regarding hypothesis no 2. During an intervention period, in which an elevated resting PPS was reduced, indicative of ANSD reversal, all the three measures of ANS function showed substantial improvements in the TTT responses. This may suggest ANSD at baseline, as well as ANSD alleviation during the intervention period. Regarding the HRV and baroreflex-mediated cardiovascular responses, these changes were only seen in BB naïve participants. In contrast, the changes in the PPS response to TTT was unaffected by this medication. Furthermore, the largest improvements in TTT responses during the intervention period were seen in BB non-users who were also PPS reverters (Tables 3, 4). This may suggest that a reduction of an elevated PPS is associated with ANSD reversal. Among PPS reverters, significant reductions were observed in resting PPS as well as in the change in PPS response to TTT during the observation period. In contrast, among PPS non-reverters, resting PPS increased and the baroreflex-medaited cardiovascilar and HRV responses to TTT did not change during the intervention period. The finding of a persistently elevated resting PPS (i.e., PPS ≥ 60 au) in non-reverters aligns with prior findings linking persistently elevated PPS to ANSD progression. Correlations between PPS and ANSD risk factors (e.g., stress, chest pain at rest, hypertension, depression) support this interpretation (Ballegaard et al., 2023; Hecquet et al., 2024). Taken together, resting PPS as well as the PPS response to TTT may represent sensitive measures for the dynamics of ANS function.

The results after one minute of TTT demonstrated significant effects on the TTT responses for the baroreflex-mediated cardiovascular responses and PPS, indicative of an acute change in the dynamics of ANS function. In response to an intervention, which reduces an elevated resting PPS as well as increasing the PPS response to TTT, the SBP response to TTT also improved, however only when BB non-users were compared to BB users (Table 3) This may represent a physiological explanation why an angina pectoris attack can be alleviated within one minute by finger-induced neuromodulation, which reduces an elevated PPS (Ballegaard et al., 2023; Hecquet et al., 2024).

The baroreflex-mediated cardiovascular response to TTT is a yardstick for a fully experimentally controllable stimulation and assessment of the dynamic functions of the sympathetic noradrenergic and parasympathetic cholinergic components of the autonomic nervous system (Jaradeh and Prieto, 2003; Freeman and Chapleau, 2013) and together with the HRV analyses they represents commonly used methods for assessing ANS function (Billman, 2013; Cygankiewicz and Zareba, 2013).

In healthy people, TTT is known to induce a rapid and transient drop in SBP combined with a compensatory increase in HR with the aim to maintain sufficient cardiac output (Silvani et al., 2017; Onizuka et al., 2015; Gabbett et al., 2001; Yokoi and Aoki, 1999; Ramirez-Marrero et al., 2008; Montano et al., 1994). The baroreflex plays an important role in this homeostatic maintenance, leading to a compensatory increase in heart rate by a rapid reduction in parasympathetic tone (White and Raven, 2014).

In the present study, the lack of correlation between HRV on the one side, and baroreflex-mediated cardiovascular response and PPS on the other side was a surprise, as HRV is a generally accepted assessment method for ANS function and ANSD (Thayer and Lane, 2007; Thayer et al., 2010; Dekker et al., 2000). In the present study we measured HRV as power spectral analysis. However, in response to ANSD reversal, measured as reduction of an elevated PPS, the changes in HRV-responses to TTT indeed were substantial. In a previous study we measured HRV as beat-to-beat variation during Stand-up test in individuals with T2D (Faber et al., 2021). Reduced beat-to-beat variation was translated into the clinical syndrome cardiovascular autonomic neuropathy (CAN), demonstrating a strong and positive correlation between PPS and the presence of CAN (Faber et al., 2021).

Thus, despite the present findings, there is an association between HRV and PPS. At baseline PPS showed stronger correlations with baroreflex than HRV (Table 5 and Figure 2). This finding, suggesting PPS as a more sensitive measure to ANS function than HRV, needs confirmation in future studies. Furthermore, the baroreflex-mediated cardiovascular response and PPS responses to TTT seem closer associated internally than those between PPS and HRV as well as those between HRV and baroreflex-mediated cardiovascular responses. This may reflect the physiological hierarchy of ANS function (Benarroch, 2020).

Beta blockade medication blocked the effect on HRV variables during the intervention period, as well as the weak association between HRV and baroreflex-mediated cardiovascular response to TTT at baseline. The additional decrease in HRV responses to TTT during the intervention period, when an elevated PPS was reduced, suggests ANSD reversal. As such, these findings confirm the association between HRV and ANS function. In the present study we observed that the HRV response to TTT seemed mainly related to parasympathetic activity, with a negative response at baseline and with further substantially decrease in the group of PPS reverters who were BB naïve (Table 3 and Figures 4, 3). This may be interpreted as magnification in the parasympathetic withdrawal during the follow-up period in response to the reduction of the elevated resting PPS.

Furthermore, the findings confirm generally accepted knowledge, that beta blockade medication influences baroreceptor sensitivity as well as HRV. The findings also confirm previous findings that PPS is not influenced by this medication (Faber et al., 2021; Ballegaard et al., 2016). As such, the findings are compatible with the hypothesis, previously stated (Faber et al., 2021; Faber et al., 2023), that the regulation of PPS may take place centrally in the ANS, and potentially by the orexin receptor system of the lateral hypothalamus.

Another aim of the study was to test clinical observation that one-minutes changes in PPS may be used as quality control for the daily applied sensory nerve stimulation of the patients and when learned, as a guide for the alleviation of an angina pectoris attack using nerve stimulation.

The present study shows that the one-minute response of PPS observed during TTT was associated with statistically significant and clinically relevant changes in the baroreceptor response to TTT. Furthermore, the one-minute PPS response to TTT increased after 3 months of intervention, and this increase was associated with a clinically relevant increase in the TTT response of SBP at 8-min recordings among BB non-users. Taken together, these findings are consistent with the hypotheses, that the acute reduction of tenderness observed by the user during sensory nerve stimulation (i.e., acute reduction of an elevated PPS) may be associated with physiologically relevant changes in ANS function, and that ANSD reversal may be associated with prevention of angina pectoris. This might have the potential to prevent and alleviate an angina attack. Further, the clinical observation by the user of a reduced tenderness during 1-min sensory nerve stimulation may serve a purpose as quality control for correct conduction of sensory nerve stimulation.

### Strengths and limitations

The strengths of this study were several: (i) the large number (*N* = 177) of participants studied; (ii) the use of an established experimental procedure with a controllable and dynamic stimulation of the ANS, without any participant-or researcher bias; (iii) and the combination of a cross-sectional data and follow-up data obtained by the use of an RCT study. Furthermore, the HRV, baroreflex-mediated cardiovascular response and the PPS measurement can be regarded as equally objective measures as the study is purely experimental and as such the participant has no idea or expectation regarding the obtained TTT responses.

One limitation of this study may be the non-randomized design with respect to the use of BB medication. The patients who used BB medication differed from the non-users with respect to gender (more males) and a higher prevalence of heart failure. The patients with heart failure were stable and up-titrated in anti-congestive medication, and in general, had a high performance, being in New York Heart Association class I-II (Class III and IV were exclusion criteria) (Ballegaard et al., 2016). Furthermore, there were no between-group differences with respect to the PPS, SBP, PRP and HRV responses to TTT at the baseline. Although the HR response to TTT at baseline was larger in BB-non-users than in BB users; we do not believe that this difference confounded the outcomes of the present study regarding comparison of BB non-users and users.

Another limitation may be that the PPS response to TTT is measured in individuals with stable IHD and that we did not include healthy people in the study for comparison. Thus, it may be questioned to what extent the observed PPS responses to TTT reflect a healthy ANS functioning or ANSD. In addressing this question, we have previously found that: (i) the PPS response to TTT at baseline in IHD patients correlated significantly to number of ANSD risk factors, including chest pain at rest, depression and hypertension; (ii) that when the number of these ANSD risk factors were reduced during three months of PPS-reducing intervention, this reduction correlated significantly to the change in PPS response to TTT during the same intervention period (Ballegaard et al., 2015); (iii) in the present study we found that the HRV, the SBP and PRP as well as the PPS responses to TTT all changed significantly at baseline. During the follow-up period, the magnitude of the TTT responses increased for PPS, for PRP, for SBP as well as for the HRV bands, suggesting that ANSD was present at baseline and ANSD restoration was obtained during the follow-up period.

It may also be regarded as a limitation that the study did not include Intention-to-treat analysis and that active/passive groups were pooled into reverters/non-reverters. However, the pooling allowed mechanistic insight. However, the RCT’s primary analysis (intention-to-treat) demonstrated a 3.1× higher likelihood of PPS reversion in the active intervention group, supporting the intervention’s efficacy (*P* = 0.0001).

### Clinical applications and perspectives

The present study supports previous findings of an association between PPS and ANS activity. In the same cohort of people with IHD, the used educational program has shown to improve 5-year survival (Ballegaard et al., 2023). In healthy people cardiovascular risk profile was improved (Salvini et al., 2023), in people with type 2 diabetes, the same educational program improved homeostatic regulation of glucose metabolism and reduced HbA1c substantially (Faber et al., 2023). Taken together, the present findings add support to a recent editorial, stating “that the use of PPS bears paramount clinical importance, both as a diagnostic tool for ANS activity and as a target for treatment” (Salvini et al., 2023).

With respect to the used intervention, the reverter subgroup’s improvements and moderate to large Cohen’s effect sizes in baroreflex-mediated cardiovascular response/HRV (particularly in BB non-users) highlight the clinical potential of PPS-guided interventions. We note, that to obtain approval for a new anti-depressive medicine, the US Federal Drug Administration requests a Cohen effect size of at least 0.3 (Khan and Brown, 2015). As such, the present intervention seems applicable for the treatment of ASND in people with ischemic heart disease.

The findings of substantial physiological changes in TTT response after 1-min provides objective evidence for the clinical observation of acutely reduced PPS during sensory nerve stimulation and supporting the clinical experience by people with IHD that reduction of an acutely elevated PPS in association with an anginal attack, alleviates the pain, and that the educational program may have a preventive potential in angina pectoris by reversal and prevention of ANSD.

As such, future studies may focus of the practical implementation of the technology for diagnostic, prevention, and treatment of ANSD.

## Conclusion

The three measures, HRV, Baroreflex-mediated cardiovascular response and PPS, using the TTT as an experimental set-up for dynamic ANS function showed significant responses in individuals with stable IHD and ANSD, the latter measured as elevated resting PPS. However, with respect to internal associations between the three, this was only present between the baroreflex-mediated cardiovascular response and PPS responses.

With the premises of reducing resting PPS as indicative of ANSD reversal, the follow-up data demonstrated ANSD reversal, however for the baroreflex-mediated cardiovascular response and HRV responses, only in beta-blocker naive participants. Maximal improvement in ANSD reversal was seen among PPS reverters, who were also non-users of BB medication. This medication blocked the ANSD reversal measured as the baroreflex-mediated cardiovascular response and HRV responses during follow-up, however not for PPS.

An acute (after 1-min) and substantial reduction in PPS in response to TTT was seen and associated with corresponding changes in the SBP response to TTT. In response to three months of follow-up, the PPS and SBP responses to TTT both increased dramatically in PPS reverters, while they were reduced in PPS non-reverters.

Thus overall, PPS is a simple, sensitive, easy to use and promising stand-alone measure for the dynamics of ANS function. Reversal of an elevated PPS was associated with clinically relevant improvement regarding the SBP and HRV responses to tilting, suggesting reversal of ANSD.

## Data Availability

The original contributions presented in the study are included in the article/supplementary material, further inquiries can be directed to the corresponding author.

## References

[ref1] Aponte-Becerra L. Novak P. (2021). Tilt test: a review. J. Clin. Neurophysiol. 38, 279–286. doi: 10.1097/WNP.0000000000000625, PMID: 34009851

[ref2] Baer L. B. M. (2010). “Understanding rating scales and assessment instruments” in Handbook of clinicla rating scales and assessment in psychiatry and mental health. ed. Rosenbaum J. F. M. (New York: Humana Press).

[ref3] Ballegaard S. Bergmann N. Karpatschof B. Kristiansen J. Gyntelberg F. Arendt-Nielsen L.. (2015). Association between pressure pain sensitivity and autonomic function as assessed by a tilt table test. Scand. J. Clin. Lab. Invest. 75, 345–354. doi: 10.3109/00365513.2015.1028095, PMID: 25833816

[ref4] Ballegaard S. Bergmann N. Karpatschof B. Kristiansen J. Gyntelberg F. Arendt-Nielsen L.. (2016). Association between depression, pressure pain sensitivity, stress and autonomous nervous system function in stable ischemic heart disease: impact of beta-adrenergic receptor blockade. J. Behav. Brain Sci. 6, 317–328. doi: 10.4236/jbbs.2016.68031

[ref5] Ballegaard S. Faber J. Selmer C. Gyntelberg F. Kreiner S. Karpatschof B.. (2023). In ischemic heart disease, reduced sensitivity to pressure at the sternum accompanies lower mortality after five years: evidence from a randomized controlled trial. J. Clin. Med. 12:7585. doi: 10.3390/jcm12247585, PMID: 38137654 PMC10744062

[ref6] Ballegaard S. Karpatschof B. Trojaborg W. Hansen A. M. Magnusson G. Petersen P. B. (2009). A simple and objective marker for stress. Scand. J. Clin. Lab. Invest. 69, 713–721. doi: 10.3109/00365510903042734, PMID: 19544223

[ref7] Ballegaard S. Petersen P. B. Harboe G. S. Karpatschof B. Gyntelberg F. Faber J. (2014). The association between changes in pressure pain sensitivity and changes in cardiovascular physiological factors associated with persistent stress. Scand. J. Clin. Lab. Invest. 74, 116–125. doi: 10.3109/00365513.2013.862847, PMID: 24313546

[ref8] Benarroch E. E. (2020). Physiology and pathophysiology of the autonomic nervous system. Continuum 26, 12–24. doi: 10.1212/CON.0000000000000817, PMID: 31996619

[ref9] Bergmann N. Ballegaard S. Bech P. Hjalmarson A. Krogh J. Gyntelberg F.. (2014). The effect of daily self-measurement of pressure pain sensitivity followed by acupressure on depression and quality of life versus treatment as usual in ischemic heart disease: a randomized clinical trial. PloS One 9:e97553. doi: 10.1371/journal.pone.0097553, PMID: 24849077 PMC4029626

[ref10] Bergmann N. Ballegaard S. Holmager P. Kristiansen J. Gyntelberg F. Andersen L. J.. (2013). Pressure pain sensitivity: a new method of stress measurement in patients with ischemic heart disease. Scand. J. Clin. Lab. Invest. 73, 373–379. doi: 10.3109/00365513.2013.785588, PMID: 23607612

[ref11] Berntson G. G. Bigger J. T. Jr. Eckberg D. L. Grossman P. Kaufmann P. G. Malik M.. (1997). Heart rate variability: origins, methods, and interpretive caveats. Psychophysiology 34, 623–648. doi: 10.1111/j.1469-8986.1997.tb02140.x, PMID: 9401419

[ref12] Billman G. E. (2013). The effect of heart rate on the heart rate variability response to autonomic interventions. Front. Physiol. 4:222. doi: 10.3389/fphys.2013.00222, PMID: 23986716 PMC3752439

[ref13] Brinza C. Floria M. Covic A. Burlacu A. (2021). Measuring heart rate variability in patients admitted with st-elevation myocardial infarction for the prediction of subsequent cardiovascular events: a systematic review. Medicina 57:1021. doi: 10.3390/medicina57101021, PMID: 34684058 PMC8540987

[ref14] Brown H. Prescott R. (2014). Applied mixed models in medicine (3rd ed.). Hoboken, NJ: John Wiley & Sons.

[ref15] Carstensen B. Kristensen J. K. Marcussen M. M. Borch-Johnsen K. (2011). The national diabetes register. Scand. J. Public Health 39, 58–61. doi: 10.1177/1403494811404278, PMID: 21775353

[ref16] Cheshire W. P. Jr. Goldstein D. S. (2019). Autonomic uprising: the tilt table test in autonomic medicine. Clin. Auton. Res. 29, 215–230. doi: 10.1007/s10286-019-00598-9, PMID: 30838497 PMC8897774

[ref17] Cygankiewicz I. Zareba W. (2013). “Chapter 31- heart rate variability” in Handbook of clinical neurology. eds. Buijs R. M. Swaab D. F., vol. 117 (Amsterdam, Netherlands: Elsevier), 379–393. 10.1016/B978-0-444-53491-0.00031-6 24095141

[ref18] Cygankiewicz I. Zareba W. (2013). Heart rate variability. Handb. Clin. Neurol. 117, 379–393. doi: 10.1016/B978-0-444-53491-0.00031-6, PMID: 24095141

[ref19] Dekker J. M. Crow R. S. Folsom A. R. Hannan P. J. Liao D. Swenne C. A.. (2000). Low heart rate variability in a 2-minute rhythm strip predicts risk of coronary heart disease and mortality from several causes: the ARIC study. Atherosclerosis Risk Commun. Circul. 102, 1239–1244. doi: 10.1161/01.CIR.102.11.1239, PMID: 10982537

[ref20] Electrophysiology, Task Force of the European Society of Cardiology the North American Society of Pacing (1996). Heart rate variability. Standards of measurement, physiological interpretation, and clinical use. Circulation 93, 1043–1065. doi: 10.1161/01.CIR.93.5.1043, PMID: 8598068

[ref21] Faber J. Ballegaard S. Ørsted N. Eldrup E. Karpatschof B. Gyntelberg F.. (2023). In type 2 diabetes mellitus, normalization of hemoglobin A1c accompanies reduced sensitivity to pressure at the sternum. Front. Neurosci. 17:1067098. doi: 10.3389/fnins.2023.1067098, PMID: 37389368 PMC10303981

[ref22] Faber J. Eldrup E. Selmer C. Pichat C. Hecquet S. K. Watt T.. (2021). Reduction of pressure pain sensitivity as novel non-pharmacological therapeutic approach to type 2 diabetes: a randomized trial. Front. Neurosci. 15:613858. doi: 10.3389/fnins.2021.613858, PMID: 33776633 PMC7991917

[ref23] Feldman D. Elton T. S. Menachemi D. M. Wexler R. K. (2010). Heart rate control with adrenergic blockade: clinical outcomes in cardiovascular medicine. Vasc. Health Risk Manag. 6:397. doi: 10.2147/VHRM.S10358, PMID: 20539841 PMC2882891

[ref24] Fleischer J. (2012). Diabetic autonomic imbalance and glycemic variability. J. Diabetes Sci. Technol. 6, 1207–1215. doi: 10.1177/193229681200600526, PMID: 23063048 PMC3570856

[ref25] Freeman R. Chapleau M. W. (2013). Testing the autonomic nervous system. Handb. Clin. Neurol. 115, 115–136. doi: 10.1016/B978-0-444-52902-2.00007-2, PMID: 23931777

[ref26] Gabbett T. J. Weston S. B. Barrett R. S. Gass G. C. (2001). Cardiovascular regulation during head-up tilt in healthy 20-30-year-old and 70-75-year-old men. Clin. Sci. 100, 199–206. doi: 10.1042/cs1000199 11171289

[ref27] Goldstein D. S. (2019). How does homeostasis happen? Integrative physiological, systems biological, and evolutionary perspectives. Am. J. Phys. Regul. Integr. Comp. Phys. 316, R301–R317. doi: 10.1152/ajpregu.00396.2018, PMID: 30649893 PMC6483214

[ref28] Gordan R. Gwathmey J. K. Xie L. H. (2015). Autonomic and endocrine control of cardiovascular function. World J. Cardiol. 7, 204–214. doi: 10.4330/wjc.v7.i4.204, PMID: 25914789 PMC4404375

[ref29] Hecquet S. K. Ballegaard S. Eldrup E. Hansen C. S. Hansen T. W. Harboe G. S.. (2024). New diabetic treatment by alleviation of autonomic nervous system dysfunction measured as periosteal pressure sensitivity at sternum improves empowerment, treatment satisfaction, and self-reported health of people with type 2 diabetes: a randomized trial. Diabetes Metabolic Syndrome Obesity 17, 2519–2531. doi: 10.2147/DMSO.S455216, PMID: 38910915 PMC11193981

[ref30] Jaradeh S. S. Prieto T. E. (2003). Evaluation of the autonomic nervous system. Phys. Med. Rehabil. Clin. N. Am. 14, 287–305. doi: 10.1016/S1047-9651(02)00121-3, PMID: 12795517

[ref31] Karemaker J. M. (2017). An introduction into autonomic nervous function. Physiol. Meas. 38, R89–r118. doi: 10.1088/1361-6579/aa6782, PMID: 28304283

[ref32] Khan A. Brown W. A. (2015). Antidepressants versus placebo in major depression: an overview. World Psychiatry 14, 294–300. doi: 10.1002/wps.20241, PMID: 26407778 PMC4592645

[ref33] Khandelwal E. Tripathi S. Gupta A. Singh A. (2023). Profile of cardiovascular autonomic dysfunctions in breast Cancer patients. Cureus. 15:e46773. doi: 10.7759/cureus.46773, PMID: 37954780 PMC10632730

[ref34] Kristiansen J. Ektor-Andersen J. Bondesson E. Ørbæk P. Persson R. Garde A. H.. (2011). Low heart rate variability is associated with extended pain-related sick leave among employed care-seekers. J. Rehabil. Med. 43, 976–982. doi: 10.2340/16501977-0882, PMID: 22031342

[ref35] Mistry E. A. Yeatts S. D. Khatri P. Mistry A. M. Detry M. Viele K.. (2022). National Institutes of Health stroke scale as an outcome in stroke research: value of ANCOVA over analyzing change from baseline. Stroke 53, e150–e155. doi: 10.1161/STROKEAHA.121.034859, PMID: 35012328 PMC8960347

[ref36] Montano N. Ruscone T. G. Porta A. Lombardi F. Pagani M. Malliani A. (1994). Power spectrum analysis of heart rate variability to assess the changes in sympathovagal balance during graded orthostatic tilt. Circulation 90, 1826–1831. doi: 10.1161/01.CIR.90.4.1826, PMID: 7923668

[ref37] Novak P. (2011). Quantitative autonomic testing. JoVE 53:e2502. doi: 10.3791/2502-v, PMID: 21788940 PMC3196175

[ref38] Onizuka C. Niimi Y. Sato M. Sugenoya J. (2015). Arterial blood pressure response to head-up tilt test and orthostatic tolerance in nurses. Environ. Health Prev. Med. 20, 262–270. doi: 10.1007/s12199-015-0455-5, PMID: 25894388 PMC4491061

[ref39] Ramirez-Marrero F. A. Charkoudian N. Hart E. C. Schroeder D. Zhong L. Eisenach J. H.. (2008). Cardiovascular dynamics in healthy subjects with differing heart rate responses to tilt. J. Appl. Physiol. (1985) 105, 1448–1453. doi: 10.1152/japplphysiol.90796.2008, PMID: 18756006 PMC2584840

[ref40] Salvini V. Accioli R. Lazzerini P. E. Acampa M. (2023). New challenges and future perspectives in autonomic neuroscience. Front. Neurosci. 17:1271499. doi: 10.3389/fnins.2023.1271499, PMID: 37680971 PMC10482394

[ref41] Silvani A. Calandra-Buonaura G. Johnson B. D. van Helmond N. Barletta G. Cecere A. G.. (2017). Physiological mechanisms mediating the coupling between heart period and arterial pressure in response to postural changes in humans. Front. Physiol. 8:163. doi: 10.3389/fphys.2017.00163, PMID: 28396638 PMC5366337

[ref42] Thayer J. F. Lane R. D. (2007). The role of vagal function in the risk for cardiovascular disease and mortality. Biol. Psychol. 74, 224–242. doi: 10.1016/j.biopsycho.2005.11.013, PMID: 17182165

[ref43] Thayer J. F. Yamamoto S. S. Brosschot J. F. (2010). The relationship of autonomic imbalance, heart rate variability and cardiovascular disease risk factors. Int. J. Cardiol. 141, 122–131. doi: 10.1016/j.ijcard.2009.09.543, PMID: 19910061

[ref44] White D. W. Raven P. B. (2014). Autonomic neural control of heart rate during dynamic exercise: revisited. J. Physiol. 592, 2491–2500. doi: 10.1113/jphysiol.2014.271858, PMID: 24756637 PMC4080933

[ref45] Yokoi Y. Aoki K. (1999). Relationship between blood pressure and heart-rate variability during graded head-up tilt. Acta Physiol. Scand. 165, 155–161. doi: 10.1046/j.1365-201x.1999.00493.x 10090326

